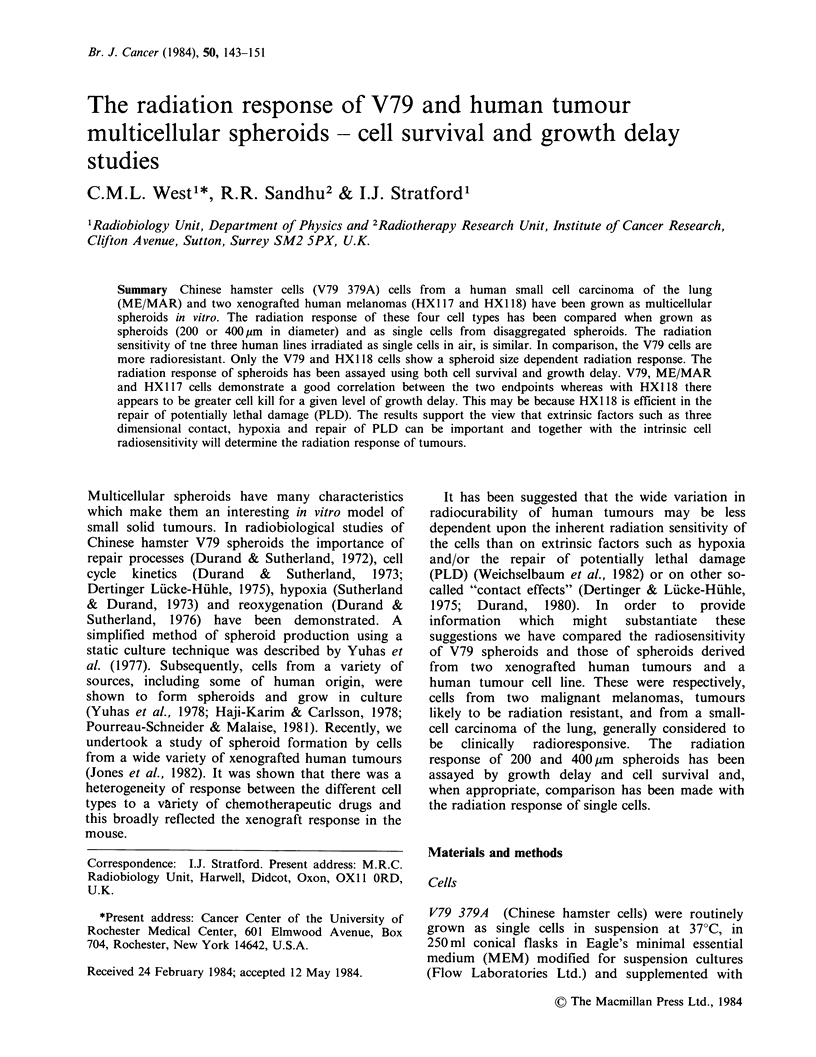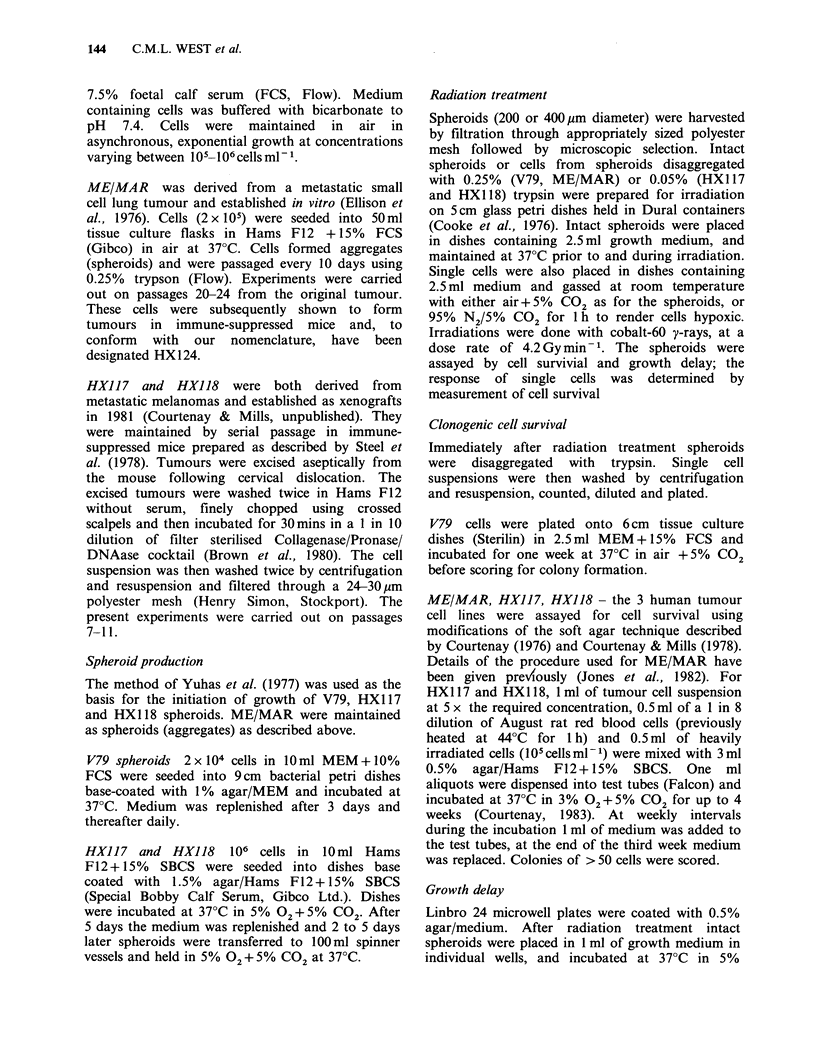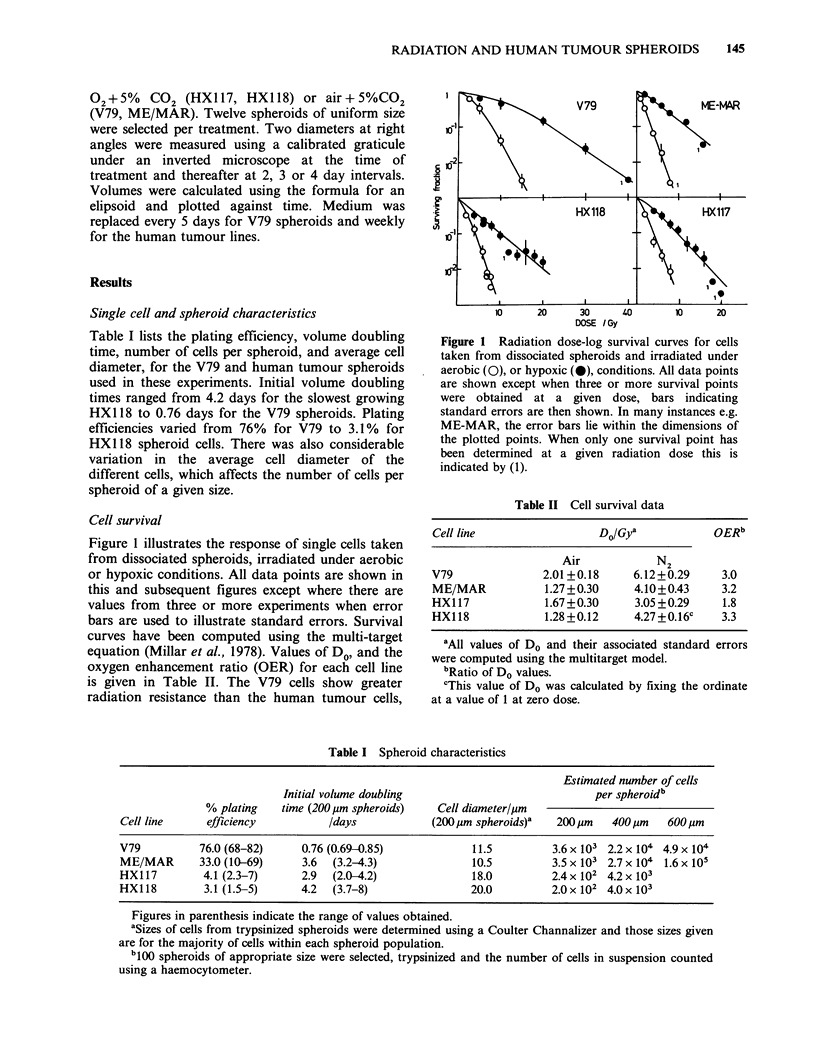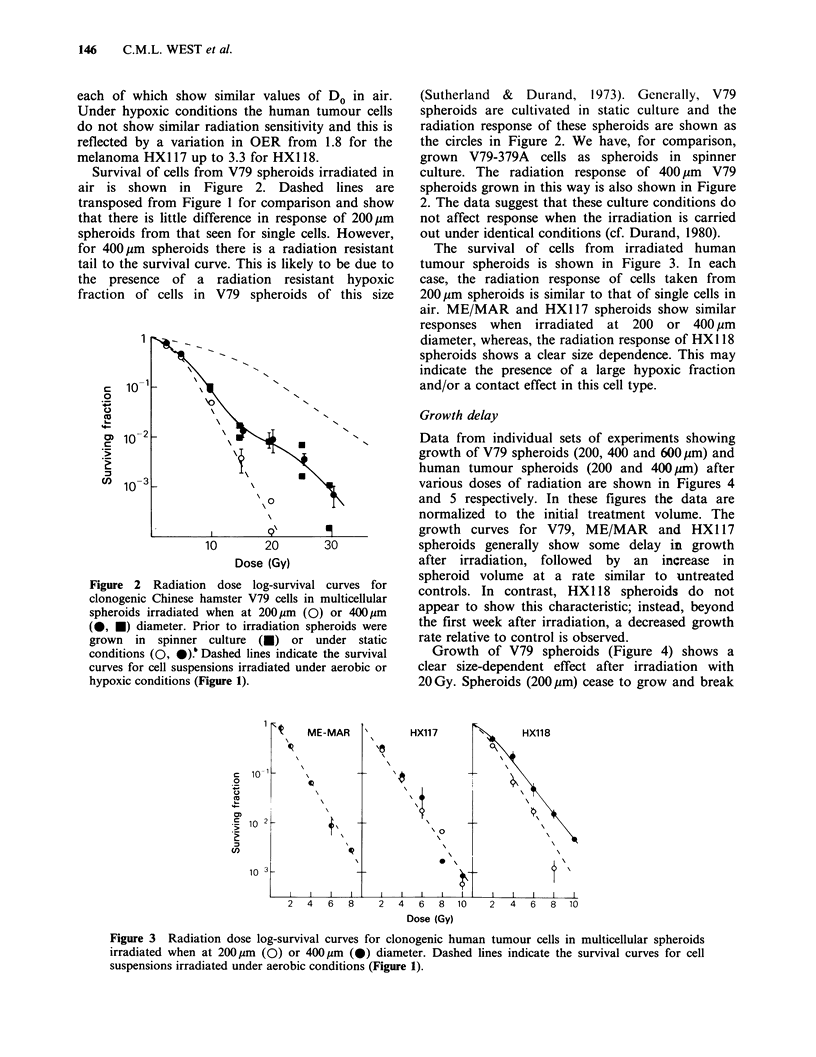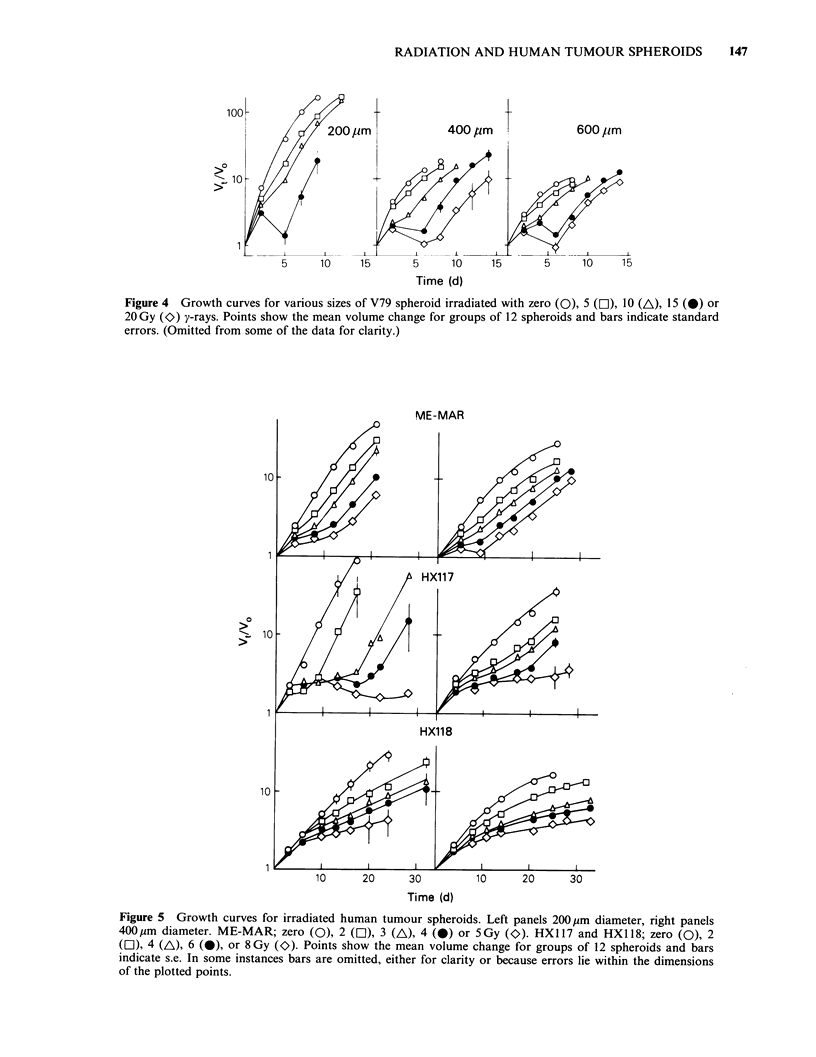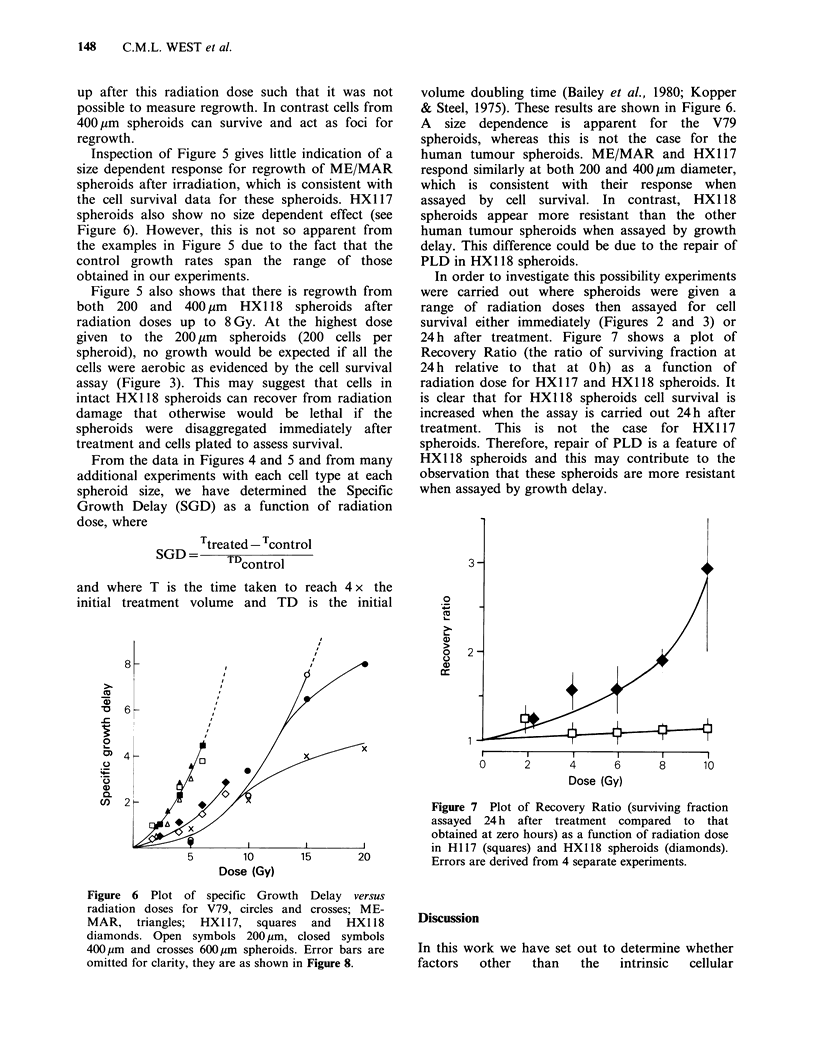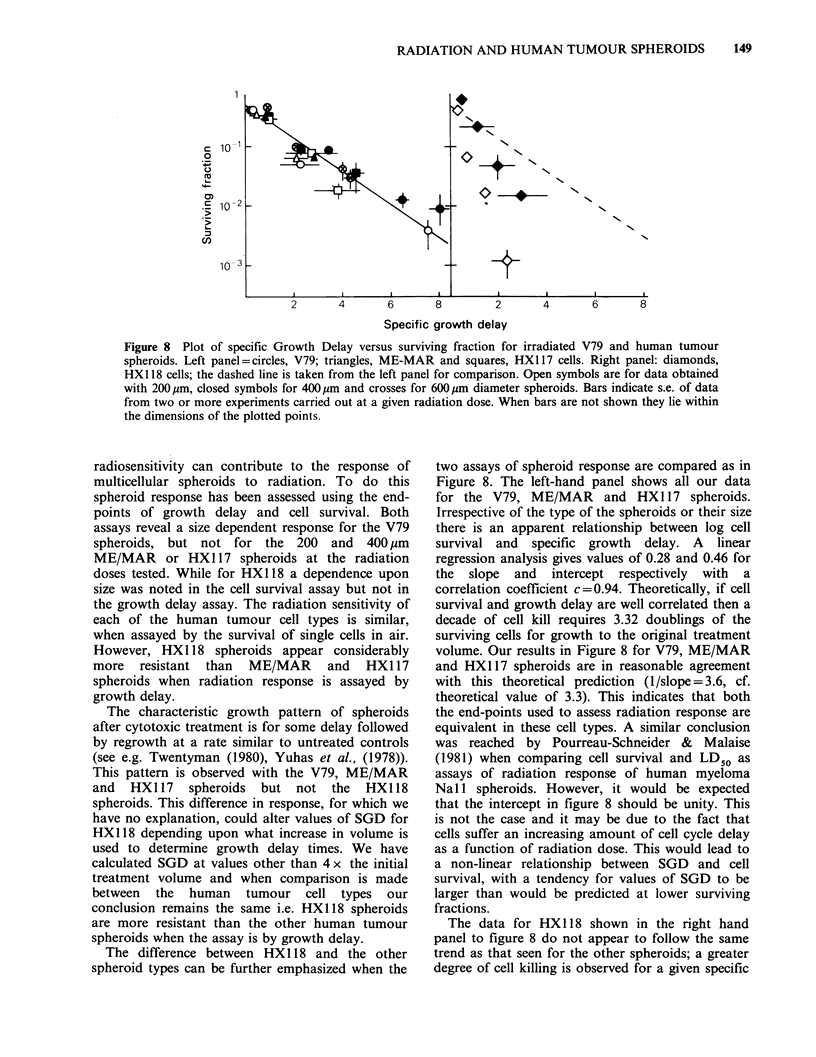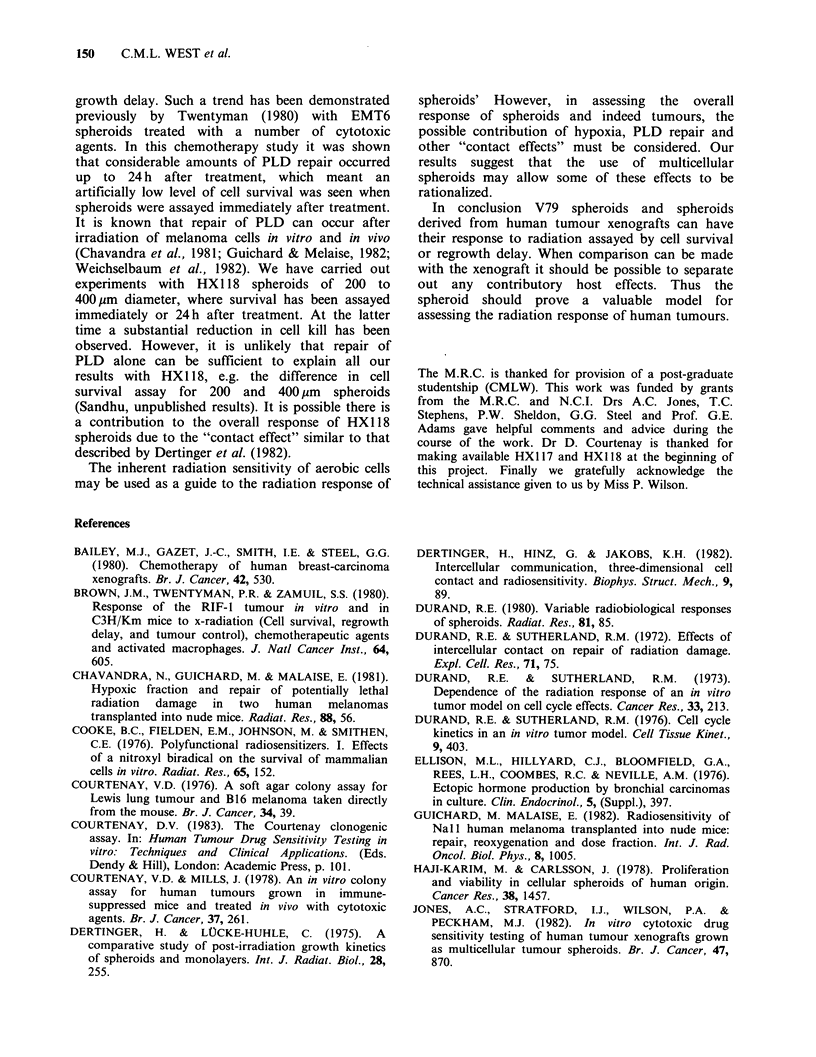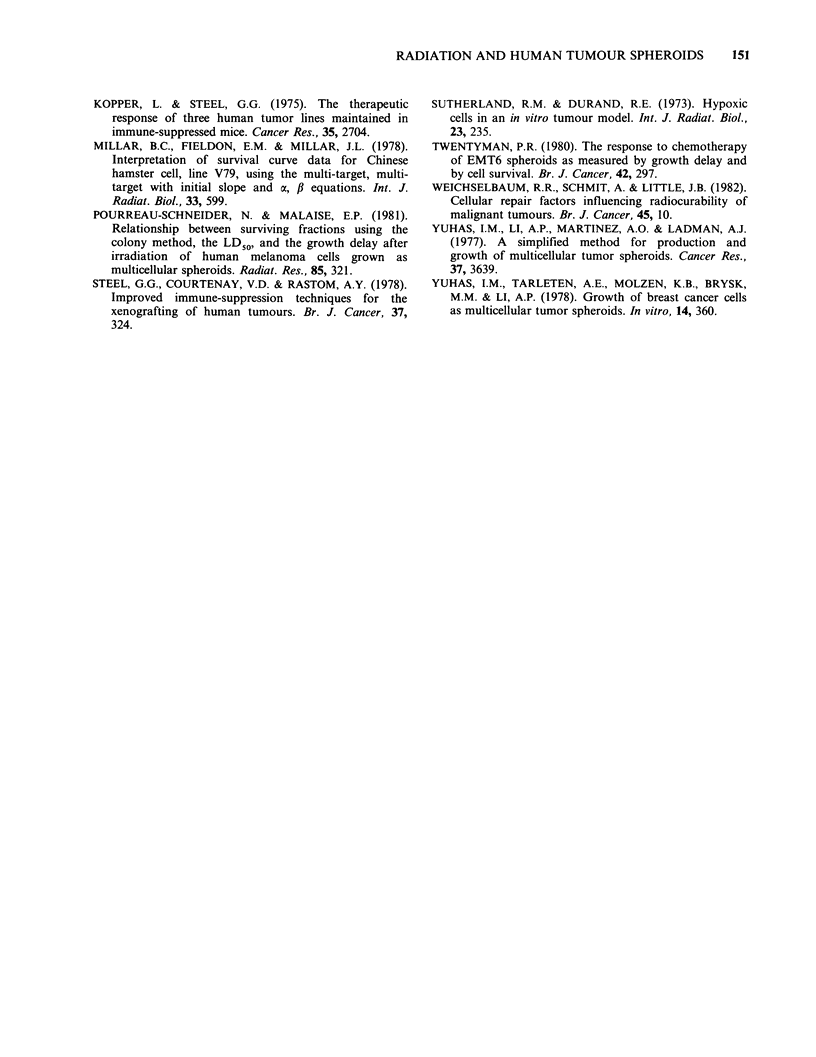# The radiation response of V79 and human tumour multicellular spheroids--cell survival and growth delay studies.

**DOI:** 10.1038/bjc.1984.156

**Published:** 1984-08

**Authors:** C. M. West, R. R. Sandhu, I. J. Stratford

## Abstract

Chinese hamster cells (V79 379A) cells from a human small cell carcinoma of the lung (ME/MAR) and two xenografted human melanomas (HX117 and HX118) have been grown as multicellular spheroids in vitro. The radiation response of these four cell types has been compared when grown as spheroids (200 or 400 micron in diameter) and as single cells from disaggregated spheroids. The radiation sensitivity of the three human lines irradiated as single cells in air, is similar. In comparison, the V79 cells are more radioresistant. Only the V79 and HX118 cells show a spheroid size dependent radiation response. The radiation response of spheroids has been assayed using both cell survival and growth delay. V79, ME/MAR and HX117 cells demonstrate a good correlation between the two endpoints whereas with HX118 there appears to be greater cell kill for a given level of growth delay. This may be because HX118 is efficient in the repair of potentially lethal damage (PLD). The results support the view that extrinsic factors such as three dimensional contact, hypoxia and repair of PLD can be important and together with the intrinsic cell radiosensitivity will determine the radiation response of tumours.


*Br. J. Cancer* (1984), **50**, 143–151

### The radiation response of V79 and human tumour multicellular spheroids – cell survival and growth delay studies

C.M.L. West[1]*, R.R. Sandhu[2] & I.J. Stratford[1]

[1]*Radiobiology Unit, Department of Physics and* [2]*Radiotherapy Research Unit, Institute of Cancer Research, Clifton Avenue, Sutton, Surrey SM2 5PX, U.K.*

**Summary** Chinese hamster cells (V79 379A) cells from a human small cell carcinoma of the lung (ME/MAR) and two xenografted human melanomas (HX117 and HX118) have been grown as multicellular spheroids *in vitro*. The radiation response of these four cell types has been compared when grown as spheroids (200 or 400 μm in diameter) and as single cells from disaggregated spheroids. The radiation sensitivity of tne three human lines irradiated as single cells in air, is similar. In comparison, the V79 cells are more radioresistant. Only the V79 and HX118 cells show a spheroid size dependent radiation response. The radiation response of spheroids has been assayed using both cell survival and growth delay. V79, ME/MAR and HX117 cells demonstrate a good correlation between the two endpoints whereas with HX118 there appears to be greater cell kill for a given level of growth delay. This may be because HX118 is efficient in the repair of potentially lethal damage (PLD). The results support the view that extrinsic factors such as three dimensional contact, hypoxia and repair of PLD can be important and together with the intrinsic cell radiosensitivity will determine the radiation response of tumours.

Multicellular spheroids have many characteristics which make them an interesting *in vitro* model of small solid tumours. In radiobiological studies of Chinese hamster V79 spheroids the importance of repair processes (Durand & Sutherland, 1972), cell cycle kinetics (Durand & Sutherland, 1973; Dertinger Lücke-Hühle, 1975), hypoxia (Sutherland & Durand, 1973) and reoxygenation (Durand & Sutherland, 1976) have been demonstrated. A simplified method of spheroid production using a static culture technique was described by Yuhas *et al.* (1977). Subsequently, cells from a variety of sources, including some of human origin, were shown to form spheroids and grow in culture (Yuhas *et al.*, 1978; Haji-Karim & Carlsson, 1978; Pourreau-Schneider & Malaise, 1981). Recently, we undertook a study of spheroid formation by cells from a wide variety of xenografted human tumours (Jones *et al.*, 1982). It was shown that there was a heterogeneity of response between the different cell types to a vâriety of chemotherapeutic drugs and this broadly reflected the xenograft response in the mouse.

Correspondence: I.J. Stratford. Present address: M.R.C. Radiobiology Unit, Harwell, Didcot, Oxon, OX11 0RD, U.K.

*Present address: Cancer Center of the University of Rochester Medical Center, 601 Elmwood Avenue, Box 704, Rochester, New York 14642, U.S.A.

Received 24 February 1984; accepted 12 May 1984.

It has been suggested that the wide variation in radiocurability of human tumours may be less dependent upon the inherent radiation sensitivity of the cells than on extrinsic factors such as hypoxia and/or the repair of potentially lethal damage (PLD) (Weichselbaum *et al.*, 1982) or on other so-called "contact effects" (Dertinger & Lücke-Hühle, 1975; Durand, 1980). In order to provide information which might substantiate these suggestions we have compared the radiosensitivity of V79 spheroids and those of spheroids derived from two xenografted human tumours and a human tumour cell line. These were respectively, cells from two malignant melanomas, tumours likely to be radiation resistant, and from a small-cell carcinoma of the lung, generally considered to be clinically radioresponsive. The radiation response of 200 and 400 μm spheroids has been assayed by growth delay and cell survival and, when appropriate, comparison has been made with the radiation response of single cells.

### Materials and methods

### *Cells*

*V79 379A* (Chinese hamster cells) were routinely grown as single cells in suspension at 37°C, in 250 ml conical flasks in Eagle's minimal essential medium (MEM) modified for suspension cultures (Flow Laboratories Ltd.) and supplemented with

© The Macmillan Press Ltd., 1984

7.5% foetal calf serum (FCS, Flow). Medium containing cells was buffered with bicarbonate to pH 7.4. Cells were maintained in air in asynchronous, exponential growth at concentrations varying between $10^5$–$10^6$ cells ml$^{-1}$.

*ME/MAR* was derived from a metastatic small cell lung tumour and established *in vitro* (Ellison *et al.*, 1976). Cells ($2\times10^5$) were seeded into 50 ml tissue culture flasks in Hams F12 +15% FCS (Gibco) in air at 37°C. Cells formed aggregates (spheroids) and were passaged every 10 days using 0.25% trypson (Flow). Experiments were carried out on passages 20–24 from the original tumour. These cells were subsequently shown to form tumours in immune-suppressed mice and, to conform with our nomenclature, have been designated HX124.

*HX117 and HX118* were both derived from metastatic melanomas and established as xenografts in 1981 (Courtenay & Mills, unpublished). They were maintained by serial passage in immune-suppressed mice prepared as described by Steel *et al.* (1978). Tumours were excised aseptically from the mouse following cervical dislocation. The excised tumours were washed twice in Hams F12 without serum, finely chopped using crossed scalpels and then incubated for 30 mins in a 1 in 10 dilution of filter sterilised Collagenase/Pronase/DNAase cocktail (Brown *et al.*, 1980). The cell suspension was then washed twice by centrifugation and resuspension and filtered through a 24–30 $\mu$m polyester mesh (Henry Simon, Stockport). The present experiments were carried out on passages 7–11.

*Spheroid production*

The method of Yuhas *et al.* (1977) was used as the basis for the initiation of growth of V79, HX117 and HX118 spheroids. ME/MAR were maintained as spheroids (aggregates) as described above.

*V79 spheroids* $2\times10^4$ cells in 10 ml MEM + 10% FCS were seeded into 9 cm bacterial petri dishes base-coated with 1% agar/MEM and incubated at 37°C. Medium was replenished after 3 days and thereafter daily.

*HX117 and HX118* $10^6$ cells in 10 ml Hams F12 + 15% SBCS were seeded into dishes base coated with 1.5% agar/Hams F12 + 15% SBCS (Special Bobby Calf Serum, Gibco Ltd.). Dishes were incubated at 37°C in 5% $O_2$ + 5% $CO_2$. After 5 days the medium was replenished and 2 to 5 days later spheroids were transferred to 100 ml spinner vessels and held in 5% $O_2$ + 5% $CO_2$ at 37°C.

*Radiation treatment*

Spheroids (200 or 400 $\mu$m diameter) were harvested by filtration through appropriately sized polyester mesh followed by microscopic selection. Intact spheroids or cells from spheroids disaggregated with 0.25% (V79, ME/MAR) or 0.05% (HX117 and HX118) trypsin were prepared for irradiation on 5 cm glass petri dishes held in Dural containers (Cooke *et al.*, 1976). Intact spheroids were placed in dishes containing 2.5 ml growth medium, and maintained at 37°C prior to and during irradiation. Single cells were also placed in dishes containing 2.5 ml medium and gassed at room temperature with either air + 5% $CO_2$ as for the spheroids, or 95% $N_2$/5% $CO_2$ for 1 h to render cells hypoxic. Irradiations were done with cobalt-60 $\gamma$-rays, at a dose rate of 4.2 Gy min$^{-1}$. The spheroids were assayed by cell survivial and growth delay; the response of single cells was determined by measurement of cell survival

*Clonogenic cell survival*

Immediately after radiation treatment spheroids were disaggregated with trypsin. Single cell suspensions were then washed by centrifugation and resuspension, counted, diluted and plated.

*V79* cells were plated onto 6 cm tissue culture dishes (Sterilin) in 2.5 ml MEM + 15% FCS and incubated for one week at 37°C in air +5% $CO_2$ before scoring for colony formation.

*ME/MAR, HX117, HX118* – the 3 human tumour cell lines were assayed for cell survival using modifications of the soft agar technique described by Courtenay (1976) and Courtenay & Mills (1978). Details of the procedure used for ME/MAR have been given previously (Jones *et al.*, 1982). For HX117 and HX118, 1 ml of tumour cell suspension at 5× the required concentration, 0.5 ml of a 1 in 8 dilution of August rat red blood cells (previously heated at 44°C for 1 h) and 0.5 ml of heavily irradiated cells ($10^5$ cells ml$^{-1}$) were mixed with 3 ml 0.5% agar/Hams F12 + 15% SBCS. One ml aliquots were dispensed into test tubes (Falcon) and incubated at 37°C in 3% $O_2$ + 5% $CO_2$ for up to 4 weeks (Courtenay, 1983). At weekly intervals during the incubation 1 ml of medium was added to the test tubes, at the end of the third week medium was replaced. Colonies of >50 cells were scored.

*Growth delay*

Linbro 24 microwell plates were coated with 0.5% agar/medium. After radiation treatment intact spheroids were placed in 1 ml of growth medium in individual wells, and incubated at 37°C in 5%

$O_2$+5% $CO_2$ (HX117, HX118) or air+5%$CO_2$ (V79, ME/MAR). Twelve spheroids of uniform size were selected per treatment. Two diameters at right angles were measured using a calibrated graticule under an inverted microscope at the time of treatment and thereafter at 2, 3 or 4 day intervals. Volumes were calculated using the formula for an elipsoid and plotted against time. Medium was replaced every 5 days for V79 spheroids and weekly for the human tumour lines.

### Results

### *Single cell and spheroid characteristics*

Table I lists the plating efficiency, volume doubling time, number of cells per spheroid, and average cell diameter, for the V79 and human tumour spheroids used in these experiments. Initial volume doubling times ranged from 4.2 days for the slowest growing HX118 to 0.76 days for the V79 spheroids. Plating efficiencies varied from 76% for V79 to 3.1% for HX118 spheroid cells. There was also considerable variation in the average cell diameter of the different cells, which affects the number of cells per spheroid of a given size.

### *Cell survival*

Figure 1 illustrates the response of single cells taken from dissociated spheroids, irradiated under aerobic or hypoxic conditions. All data points are shown in this and subsequent figures except where there are values from three or more experiments when error bars are used to illustrate standard errors. Survival curves have been computed using the multi-target equation (Millar *et al.*, 1978). Values of $D_0$, and the oxygen enhancement ratio (OER) for each cell line is given in Table II. The V79 cells show greater radiation resistance than the human tumour cells,

**Figure 1** Radiation dose-log survival curves for cells taken from dissociated spheroids and irradiated under aerobic (○), or hypoxic (●), conditions. All data points are shown except when three or more survival points were obtained at a given dose, bars indicating standard errors are then shown. In many instances e.g. ME-MAR, the error bars lie within the dimensions of the plotted points. When only one survival point has been determined at a given radiation dose this is indicated by (1).

**Table II** Cell survival data

| *Cell line* | $D_0/Gy$[a] | | *OER*[b] |
|---|---|---|---|
| | Air | $N_2$ | |
| V79 | 2.01±0.18 | 6.12±0.29 | 3.0 |
| ME/MAR | 1.27±0.30 | 4.10±0.43 | 3.2 |
| HX117 | 1.67±0.30 | 3.05±0.29 | 1.8 |
| HX118 | 1.28±0.12 | 4.27±0.16[c] | 3.3 |

[a]All values of $D_0$ and their associated standard errors were computed using the multitarget model.
[b]Ratio of $D_0$ values.
[c]This value of $D_0$ was calculated by fixing the ordinate at a value of 1 at zero dose.

**Table I** Spheroid characteristics

| *Cell line* | *% plating efficiency* | *Initial volume doubling time (200 μm spheroids) /days* | *Cell diameter/μm (200 μm spheroids)*[a] | *Estimated number of cells per spheroid*[b] 200 μm | 400 μm | 600 μm |
|---|---|---|---|---|---|---|
| V79 | 76.0 (68–82) | 0.76 (0.69–0.85) | 11.5 | $3.6\times10^3$ | $2.2\times10^4$ | $4.9\times10^4$ |
| ME/MAR | 33.0 (10–69) | 3.6 (3.2–4.3) | 10.5 | $3.5\times10^3$ | $2.7\times10^4$ | $1.6\times10^5$ |
| HX117 | 4.1 (2.3–7) | 2.9 (2.0–4.2) | 18.0 | $2.4\times10^2$ | $4.2\times10^3$ | |
| HX118 | 3.1 (1.5–5) | 4.2 (3.7–8) | 20.0 | $2.0\times10^2$ | $4.0\times10^3$ | |

Figures in parenthesis indicate the range of values obtained.
[a]Sizes of cells from trypsinized spheroids were determined using a Coulter Channalizer and those sizes given are for the majority of cells within each spheroid population.
[b]100 spheroids of appropriate size were selected, trypsinized and the number of cells in suspension counted using a haemocytometer.

each of which show similar values of $D_0$ in air. Under hypoxic conditions the human tumour cells do not show similar radiation sensitivity and this is reflected by a variation in OER from 1.8 for the melanoma HX117 up to 3.3 for HX118.

Survival of cells from V79 spheroids irradiated in air is shown in Figure 2. Dashed lines are transposed from Figure 1 for comparison and show that there is little difference in response of 200 μm spheroids from that seen for single cells. However, for 400 μm spheroids there is a radiation resistant tail to the survival curve. This is likely to be due to the presence of a radiation resistant hypoxic fraction of cells in V79 spheroids of this size

**Figure 2** Radiation dose log-survival curves for clonogenic Chinese hamster V79 cells in multicellular spheroids irradiated when at 200 μm (○) or 400 μm (●, ■) diameter. Prior to irradiation spheroids were grown in spinner culture (■) or under static conditions (○, ●). Dashed lines indicate the survival curves for cell suspensions irradiated under aerobic or hypoxic conditions (**Figure 1**).

(Sutherland & Durand, 1973). Generally, V79 spheroids are cultivated in static culture and the radiation response of these spheroids are shown as the circles in Figure 2. We have, for comparison, grown V79-379A cells as spheroids in spinner culture. The radiation response of 400 μm V79 spheroids grown in this way is also shown in Figure 2. The data suggest that these culture conditions do not affect response when the irradiation is carried out under identical conditions (cf. Durand, 1980).

The survival of cells from irradiated human tumour spheroids is shown in Figure 3. In each case, the radiation response of cells taken from 200 μm spheroids is similar to that of single cells in air. ME/MAR and HX117 spheroids show similar responses when irradiated at 200 or 400 μm diameter, whereas, the radiation response of HX118 spheroids shows a clear size dependence. This may indicate the presence of a large hypoxic fraction and/or a contact effect in this cell type.

*Growth delay*

Data from individual sets of experiments showing growth of V79 spheroids (200, 400 and 600 μm) and human tumour spheroids (200 and 400 μm) after various doses of radiation are shown in Figures 4 and 5 respectively. In these figures the data are normalized to the initial treatment volume. The growth curves for V79, ME/MAR and HX117 spheroids generally show some delay in growth after irradiation, followed by an increase in spheroid volume at a rate similar to untreated controls. In contrast, HX118 spheroids do not appear to show this characteristic; instead, beyond the first week after irradiation, a decreased growth rate relative to control is observed.

Growth of V79 spheroids (Figure 4) shows a clear size-dependent effect after irradiation with 20 Gy. Spheroids (200 μm) cease to grow and break

**Figure 3** Radiation dose log-survival curves for clonogenic human tumour cells in multicellular spheroids irradiated when at 200 μm (○) or 400 μm (●) diameter. Dashed lines indicate the survival curves for cell suspensions irradiated under aerobic conditions (**Figure 1**).

**Figure 4** Growth curves for various sizes of V79 spheroid irradiated with zero (○), 5 (□), 10 (△), 15 (●) or 20 Gy (◇) γ-rays. Points show the mean volume change for groups of 12 spheroids and bars indicate standard errors. (Omitted from some of the data for clarity.)

**Figure 5** Growth curves for irradiated human tumour spheroids. Left panels 200 μm diameter, right panels 400 μm diameter. ME-MAR; zero (○), 2 (□), 3 (△), 4 (●) or 5 Gy (◇). HX117 and HX118; zero (○), 2 (□), 4 (△), 6 (●), or 8 Gy (◇). Points show the mean volume change for groups of 12 spheroids and bars indicate s.e. In some instances bars are omitted, either for clarity or because errors lie within the dimensions of the plotted points.

up after this radiation dose such that it was not possible to measure regrowth. In contrast cells from 400 μm spheroids can survive and act as foci for regrowth.

Inspection of Figure 5 gives little indication of a size dependent response for regrowth of ME/MAR spheroids after irradiation, which is consistent with the cell survival data for these spheroids. HX117 spheroids also show no size dependent effect (see Figure 6). However, this is not so apparent from the examples in Figure 5 due to the fact that the control growth rates span the range of those obtained in our experiments.

Figure 5 also shows that there is regrowth from both 200 and 400 μm HX118 spheroids after radiation doses up to 8 Gy. At the highest dose given to the 200 μm spheroids (200 cells per spheroid), no growth would be expected if all the cells were aerobic as evidenced by the cell survival assay (Figure 3). This may suggest that cells in intact HX118 spheroids can recover from radiation damage that otherwise would be lethal if the spheroids were disaggregated immediately after treatment and cells plated to assess survival.

From the data in Figures 4 and 5 and from many additional experiments with each cell type at each spheroid size, we have determined the Specific Growth Delay (SGD) as a function of radiation dose, where

$$SGD = \frac{^{T}treated - {}^{T}control}{^{TD}control}$$

and where T is the time taken to reach 4× the initial treatment volume and TD is the initial

**Figure 6** Plot of specific Growth Delay *versus* radiation doses for V79, circles and crosses; ME-MAR, triangles; HX117, squares and HX118 diamonds. Open symbols 200 μm, closed symbols 400 μm and crosses 600 μm spheroids. Error bars are omitted for clarity, they are as shown in **Figure 8**.

volume doubling time (Bailey *et al.,* 1980; Kopper & Steel, 1975). These results are shown in Figure 6. A size dependence is apparent for the V79 spheroids, whereas this is not the case for the human tumour spheroids. ME/MAR and HX117 respond similarly at both 200 and 400 μm diameter, which is consistent with their response when assayed by cell survival. In contrast, HX118 spheroids appear more resistant than the other human tumour spheroids when assayed by growth delay. This difference could be due to the repair of PLD in HX118 spheroids.

In order to investigate this possibility experiments were carried out where spheroids were given a range of radiation doses then assayed for cell survival either immediately (Figures 2 and 3) or 24 h after treatment. Figure 7 shows a plot of Recovery Ratio (the ratio of surviving fraction at 24 h relative to that at 0 h) as a function of radiation dose for HX117 and HX118 spheroids. It is clear that for HX118 spheroids cell survival is increased when the assay is carried out 24 h after treatment. This is not the case for HX117 spheroids. Therefore, repair of PLD is a feature of HX118 spheroids and this may contribute to the observation that these spheroids are more resistant when assayed by growth delay.

**Figure 7** Plot of Recovery Ratio (surviving fraction assayed 24 h after treatment compared to that obtained at zero hours) as a function of radiation dose in H117 (squares) and HX118 spheroids (diamonds). Errors are derived from 4 separate experiments.

### Discussion

In this work we have set out to determine whether factors other than the intrinsic cellular

**Figure 8** Plot of specific Growth Delay versus surviving fraction for irradiated V79 and human tumour spheroids. Left panel = circles, V79; triangles, ME-MAR and squares, HX117 cells. Right panel: diamonds, HX118 cells; the dashed line is taken from the left panel for comparison. Open symbols are for data obtained with 200 $\mu$m, closed symbols for 400 $\mu$m and crosses for 600 $\mu$m diameter spheroids. Bars indicate s.e. of data from two or more experiments carried out at a given radiation dose. When bars are not shown they lie within the dimensions of the plotted points.

radiosensitivity can contribute to the response of multicellular spheroids to radiation. To do this spheroid response has been assessed using the end-points of growth delay and cell survival. Both assays reveal a size dependent response for the V79 spheroids, but not for the 200 and 400 $\mu$m ME/MAR or HX117 spheroids at the radiation doses tested. While for HX118 a dependence upon size was noted in the cell survival assay but not in the growth delay assay. The radiation sensitivity of each of the human tumour cell types is similar, when assayed by the survival of single cells in air. However, HX118 spheroids appear considerably more resistant than ME/MAR and HX117 spheroids when radiation response is assayed by growth delay.

The characteristic growth pattern of spheroids after cytotoxic treatment is for some delay followed by regrowth at a rate similar to untreated controls (see e.g. Twentyman (1980), Yuhas *et al.*, (1978)). This pattern is observed with the V79, ME/MAR and HX117 spheroids but not the HX118 spheroids. This difference in response, for which we have no explanation, could alter values of SGD for HX118 depending upon what increase in volume is used to determine growth delay times. We have calculated SGD at values other than 4 × the initial treatment volume and when comparison is made between the human tumour cell types our conclusion remains the same i.e. HX118 spheroids are more resistant than the other human tumour spheroids when the assay is by growth delay.

The difference between HX118 and the other spheroid types can be further emphasized when the two assays of spheroid response are compared as in Figure 8. The left-hand panel shows all our data for the V79, ME/MAR and HX117 spheroids. Irrespective of the type of the spheroids or their size there is an apparent relationship between log cell survival and specific growth delay. A linear regression analysis gives values of 0.28 and 0.46 for the slope and intercept respectively with a correlation coefficient $c = 0.94$. Theoretically, if cell survival and growth delay are well correlated then a decade of cell kill requires 3.32 doublings of the surviving cells for growth to the original treatment volume. Our results in Figure 8 for V79, ME/MAR and HX117 spheroids are in reasonable agreement with this theoretical prediction (1/slope = 3.6, cf. theoretical value of 3.3). This indicates that both the end-points used to assess radiation response are equivalent in these cell types. A similar conclusion was reached by Pourreau-Schneider & Malaise (1981) when comparing cell survival and $LD_{50}$ as assays of radiation response of human myeloma Na11 spheroids. However, it would be expected that the intercept in figure 8 should be unity. This is not the case and it may be due to the fact that cells suffer an increasing amount of cell cycle delay as a function of radiation dose. This would lead to a non-linear relationship between SGD and cell survival, with a tendency for values of SGD to be larger than would be predicted at lower surviving fractions.

The data for HX118 shown in the right hand panel to figure 8 do not appear to follow the same trend as that seen for the other spheroids; a greater degree of cell killing is observed for a given specific growth delay. Such a trend has been demonstrated previously by Twentyman (1980) with EMT6 spheroids treated with a number of cytotoxic agents. In this chemotherapy study it was shown that considerable amounts of PLD repair occurred up to 24 h after treatment, which meant an artificially low level of cell survival was seen when spheroids were assayed immediately after treatment. It is known that repair of PLD can occur after irradiation of melanoma cells *in vitro* and *in vivo* (Chavandra *et al.*, 1981; Guichard & Melaise, 1982; Weichselbaum *et al.*, 1982). We have carried out experiments with HX118 spheroids of 200 to 400 $\mu$m diameter, where survival has been assayed immediately or 24 h after treatment. At the latter time a substantial reduction in cell kill has been observed. However, it is unlikely that repair of PLD alone can be sufficient to explain all our results with HX118, e.g. the difference in cell survival assay for 200 and 400 $\mu$m spheroids (Sandhu, unpublished results). It is possible there is a contribution to the overall response of HX118 spheroids due to the "contact effect" similar to that described by Dertinger *et al.* (1982).

The inherent radiation sensitivity of aerobic cells may be used as a guide to the radiation response of spheroids' However, in assessing the overall response of spheroids and indeed tumours, the possible contribution of hypoxia, PLD repair and other "contact effects" must be considered. Our results suggest that the use of multicellular spheroids may allow some of these effects to be rationalized.

In conclusion V79 spheroids and spheroids derived from human tumour xenografts can have their response to radiation assayed by cell survival or regrowth delay. When comparison can be made with the xenograft it should be possible to separate out any contributory host effects. Thus the spheroid should prove a valuable model for assessing the radiation response of human tumours.

The M.R.C. is thanked for provision of a post-graduate studentship (CMLW). This work was funded by grants from the M.R.C. and N.C.I. Drs A.C. Jones, T.C. Stephens, P.W. Sheldon, G.G. Steel and Prof. G.E. Adams gave helpful comments and advice during the course of the work. Dr D. Courtenay is thanked for making available HX117 and HX118 at the beginning of this project. Finally we gratefully acknowledge the technical assistance given to us by Miss P. Wilson.

### References

BAILEY, M.J., GAZET, J.-C., SMITH, I.E. & STEEL, G.G. (1980). Chemotherapy of human breast-carcinoma xenografts. *Br. J. Cancer*, **42**, 530.

BROWN, J.M., TWENTYMAN, P.R. & ZAMUIL, S.S. (1980). Response of the RIF-1 tumour *in vitro* and in C3H/Km mice to x-radiation (Cell survival, regrowth delay, and tumour control), chemotherapeutic agents and activated macrophages. *J. Natl Cancer Inst.*, **64**, 605.

CHAVANDRA, N., GUICHARD, M. & MALAISE, E. (1981). Hypoxic fraction and repair of potentially lethal radiation damage in two human melanomas transplanted into nude mice. *Radiat. Res.*, **88**, 56.

COOKE, B.C., FIELDEN, E.M., JOHNSON, M. & SMITHEN, C.E. (1976). Polyfunctional radiosensitizers. I. Effects of a nitroxyl biradical on the survival of mammalian cells *in vitro*. *Radiat. Res.*, **65**, 152.

COURTENAY, V.D. (1976). A soft agar colony assay for Lewis lung tumour and B16 melanoma taken directly from the mouse. *Br. J. Cancer*, **34**, 39.

COURTENAY, D.V. (1983). The Courtenay clonogenic assay. In: *Human Tumour Drug Sensitivity Testing in vitro: Techniques and Clinical Applications*. (Eds. Dendy & Hill), London: Academic Press, p. 101.

COURTENAY, V.D. & MILLS, J. (1978). An *in vitro* colony assay for human tumours grown in immune-suppressed mice and treated *in vivo* with cytotoxic agents. *Br. J. Cancer*, **37**, 261.

DERTINGER, H. & LÜCKE-HUHLE, C. (1975). A comparative study of post-irradiation growth kinetics of spheroids and monolayers. *Int. J. Radiat. Biol.*, **28**, 255.

DERTINGER, H., HINZ, G. & JAKOBS, K.H. (1982). Intercellular communication, three-dimensional cell contact and radiosensitivity. *Biophys. Struct. Mech.*, **9**, 89.

DURAND, R.E. (1980). Variable radiobiological responses of spheroids. *Radiat. Res.*, **81**, 85.

DURAND, R.E. & SUTHERLAND, R.M. (1972). Effects of intercellular contact on repair of radiation damage. *Expl. Cell. Res.*, **71**, 75.

DURAND, R.E. & SUTHERLAND, R.M. (1973). Dependence of the radiation response of an *in vitro* tumor model on cell cycle effects. *Cancer Res.*, **33**, 213.

DURAND, R.E. & SUTHERLAND, R.M. (1976). Cell cycle kinetics in an *in vitro* tumor model. *Cell Tissue Kinet.*, **9**, 403.

ELLISON, M.L., HILLYARD, C.J., BLOOMFIELD, G.A., REES, L.H., COOMBES, R.C. & NEVILLE, A.M. (1976). Ectopic hormone production by bronchial carcinomas in culture. *Clin. Endocrinol.*, **5**, (Suppl.), 397.

GUICHARD, M. MALAISE, E. (1982). Radiosensitivity of Na11 human melanoma transplanted into nude mice: repair, reoxygenation and dose fraction. *Int. J. Rad. Oncol. Biol. Phys.*, **8**, 1005.

HAJI-KARIM, M. & CARLSSON, J. (1978). Proliferation and viability in cellular spheroids of human origin. *Cancer Res.*, **38**, 1457.

JONES, A.C., STRATFORD, I.J., WILSON, P.A. & PECKHAM, M.J. (1982). *In vitro* cytotoxic drug sensitivity testing of human tumour xenografts grown as multicellular tumour spheroids. *Br. J. Cancer*, **47**, 870.

KOPPER, L. & STEEL, G.G. (1975). The therapeutic response of three human tumor lines maintained in immune-suppressed mice. *Cancer Res.*, **35**, 2704.

MILLAR, B.C., FIELDON, E.M. & MILLAR, J.L. (1978). Interpretation of survival curve data for Chinese hamster cell, line V79, using the multi-target, multi-target with initial slope and $\alpha$, $\beta$ equations. *Int. J. Radiat. Biol.*, **33**, 599.

POURREAU-SCHNEIDER, N. & MALAISE, E.P. (1981). Relationship between surviving fractions using the colony method, the $LD_{50}$, and the growth delay after irradiation of human melanoma cells grown as multicellular spheroids. *Radiat. Res.*, **85**, 321.

STEEL, G.G., COURTENAY, V.D. & RASTOM, A.Y. (1978). Improved immune-suppression techniques for the xenografting of human tumours. *Br. J. Cancer*, **37**, 324.

SUTHERLAND, R.M. & DURAND, R.E. (1973). Hypoxic cells in an *in vitro* tumour model. *Int. J. Radiat. Biol.*, **23**, 235.

TWENTYMAN, P.R. (1980). The response to chemotherapy of EMT6 spheroids as measured by growth delay and by cell survival. *Br. J. Cancer*, **42**, 297.

WEICHSELBAUM, R.R., SCHMIT, A. & LITTLE, J.B. (1982). Cellular repair factors influencing radiocurability of malignant tumours. *Br. J. Cancer*, **45**, 10.

YUHAS, I.M., LI, A.P., MARTINEZ, A.O. & LADMAN, A.J. (1977). A simplified method for production and growth of multicellular tumor spheroids. *Cancer Res.*, **37**, 3639.

YUHAS, I.M., TARLETEN, A.E., MOLZEN, K.B., BRYSK, M.M. & LI, A.P. (1978). Growth of breast cancer cells as multicellular tumor spheroids. *In vitro*, **14**, 360.